# Notes and Notices

**Published:** 1884-06

**Authors:** 


### NOTES AND NOTICES.

Europe has no "Code."—It must appear strange to foreign eyes that American physicians are the only ones throughout the world who need a written "Code."

Woman's Homœopathic Association of Pennsylvania opened a general and maternity hospital at Nos. 2125 and 2127 North Twentieth Street on Wednesday, March 12th, 1884.

Worn-out Philosophy.—We have heard recently of a student in a homœopathic college who complained bitterly because he was compelled to study the *Organon.* He did not "want to be bothered with the worn-out philosophy of Hahnemann."

Homœopathy in the Garfield Memorial Hospital.—There is a question as to whether the homœopathists shall have any share in the new Garfield Hospital. The homœopathic sentiment is so strong in Washington that it is probable one ward of the hospital, if no more, will be turned over to the homœopathic doctors.—*Exchange.*

Hoffman's Prescription for Longevity.—The celebrated but humorous German physiologist, Hoffman, summarizes the means of reaching a great age as follows: "Avoid excess in everything, respect old habits, even bad ones; breathe a pure air, adapt your food to your temperament, shun medicines and doctors, keep a quiet conscience, a gay heart, a contented mind."

Penetrating Power of Sewer Gas.—A recent writer on sewer gas points out that asphyxia, typhoid fever, diphtheria, and scarlatina are all frequently caused by sewer gas, and as it has been demonstrated that gases will pass through brick and stone and unglazed earthenware, even against a resisting pressure of two and a half feet of water, it can hardly help becoming a question by and by whether or not a good many of our modern improvements in dwelling-houses will not have to be turned out-of-doors or improved beyond anything yet dreamed of.—*Exchange.*

Work of Worthless Colleges.—The depreciated value of an American medical diploma is a reproach to the profession, and it is therefore high time that the conferring of degrees should be entirely divorced from the department of instruction in medical colleges. This opinion is fully warranted by my own experience as a medical examiner. Not long ago a young gentleman—a graduate of a medical college in "good standing"—came to me for registration, and, to my utter astonishment, he could not answer the question, "What is sanitary science?" Another graduate in medicine, when asked "What is semeiology?" answered, "A description of the *spermatozoa.*"—J. E. Reeves, M. D.

